# Open Spinal Dysraphism Without Hindbrain Herniation—Natural History and Postnatal Outcome

**DOI:** 10.1002/pd.6855

**Published:** 2025-07-08

**Authors:** I. Bedei, C. C. Kik, R. Axt‐Fliedner, P. L. J. DeKoninck, W. Ventura, S. Köhler, M. Schulze, T. Struffert, M. Kolodziej, D. Diehl, B. Sass, J. K. H. Spoor, C. Keil

**Affiliations:** ^1^ Department of Prenatal Medicine and Fetal Therapy Justus‐Liebig University Giessen Giessen Germany; ^2^ Department of Obstetrics and Gynaecology Division of Obstetrics and Fetal Medicine Erasmus MC Sophia Children's Hospital University Medical Center Rotterdam the Netherlands; ^3^ Fetal Medicine Unit at Medicina Fetal Peru & Instituto Nacional Materno‐Perinatal Lima Peru; ^4^ Department of Prenatal Medicine and Fetal Therapy Philipps University Marburg Germany; ^5^ Department of Neuroradiology Philipps University Marburg Germany; ^6^ Department of Neuroradiology Justus‐Liebig University Giessen Germany; ^7^ Department of Neurosurgery Justus‐Liebig University Giessen Germany; ^8^ Department of Pediatric Neurology Justus‐Liebig University Giessen Giessen Germany; ^9^ Department of Neurosurgery Philipps University Marburg Germany; ^10^ Department of Pediatric Neurosurgery Erasmus MC Sophia Children's Hospital University Medical Center Rotterdam the Netherlands

## Abstract

**Objective:**

To report the natural history of fetuses with open spinal dysraphism (OSD) without hindbrain herniation (HBH) during second‐trimester evaluation.

**Methods:**

A multicenter retrospective cohort study was conducted across three prenatal centers to evaluate fetuses with OSD. We reviewed cases with postnatally confirmed OSD without prenatal HBH at 19–27 weeks. Standardized prenatal evaluation consisted of repetitive ultrasound and magnetic resonance imaging. Postnatal outcome measures involved imaging, intraoperative findings and neurological function tests.

**Results:**

Among 280 fetuses with OSD, evaluated at a median gestational age of 21 weeks, a total of 12 (4%) lacked HBH. Moderate ventriculomegaly was observed in 33% of cases without HBH. Corpus callosum anomalies were not detected. Postnatally, HBH was present in 50%, while the shunt rate remained low (20%). In 80%, postnatal motor function (MF) was equal to or better based on the anatomical level. In 33%, MF after birth declined in comparison to the first fetal functional assessment in the second trimester.

**Conclusion:**

Fetuses with OSD and absent HBH in the second trimester demonstrate a low postnatal shunt rate. MF was frequently impaired at the initial second‐trimester assessment, and in about a third of cases, postnatal MF seemed to have worsened. These findings may inform counseling and question the place of fetal surgery for this subgroup.

## Summary

- What is already known about the topic?
  ◦ Hindbrain herniation (HBH) is a characteristic feature of open spinal dysraphism (OSD) and a mandatory condition for prenatal surgery in most centers. Only a limited body of research has been conducted on fetuses with OSD without HBH. The natural history and postnatal outcomes of these cases remain poorly understood.
- What does this study add?
  ◦ A subset of fetuses with OSD demonstrates absence of HBH between 19 and 27 weeks of gestation, predominantly in cases of myelomeningocele (MMC) rather than myeloschisis (MS). Severe ventriculomegaly is rare within this group. Although HBH may develop later in gestation, the requirement for cerebrospinal fluid shunting remains low. Motor function may be compromised already in the second trimester.

## 1 Introduction

The Management of Myelomeningocele Study (MOMS) demonstrated the clinical benefits of prenatal surgery for fetuses diagnosed with myelomeningocele (MMC) or myeloschisis (MS), including a significant reduction in the need for shunt placement for hydrocephalus [1]. Additionally, prenatal repair mitigates the “second hit” phenomenon — a progressive secondary injury to the exposed and non‐neurulated neural tissue (placode). This ongoing damage, driven by exposure of the placode to the intrauterine environment, contributes to functional deterioration throughout pregnancy, surpassing the primary structural malformation in its impact [2, 3]. One of the principal eligibility criteria for inclusion in the MOMS trial was the presence of hindbrain herniation (HBH), a hallmark of Chiari II malformation, which occurs in nearly all cases of open spinal dysraphism [1]. HBH is thought to originate early in fetal development due to cerebrospinal fluid (CSF) leakage and inadequate expansion of the posterior fossa, contributing to abnormal CSF flow and ventricular enlargement [4, 5, 6]. HBH is known to affect motor function, cranial nerve integrity, and cognitive development, and may also lead to more severe brainstem‐related complications such as apnoea, neurogenic dysphagia, aspiration, and vocal cord paralysis [7, 8, 9, 10].

Prenatal surgical intervention typically leads to the regression of HBH before birth, restoring posterior fossa anatomy and CSF dynamics, as evidenced by improvements in the clivus‐supraocciput angle (CSA) [11, 12]. Prenatal repair has been associated with a lower incidence of posterior fossa anomalies, brainstem kinking, and tectal beaking, and a reduced risk of severe brainstem dysfunction during childhood [13, 14, 15]. A subset of fetuses with MMC may present without HBH during prenatal evaluation [16]. Historically, these cases have been excluded from prenatal surgery [1]. While the neural tissue remains exposed in such cases, the natural progression of HBH in the absence of early signs remains unclear, and the need for postnatal CSF diversion is largely unknown [17]. Nonetheless, some centers argue for reconsidering exclusion criteria for fetal therapy, raising ethical concerns about withholding treatment based on strict protocol definitions [18, 19]. Recently, a study has explored these fetuses' natural history and postnatal outcomes [9].

Our study aims to contribute additional insights into the natural history and associated findings of these lesions to improve prenatal counseling and support clinical decision‐making.

## 2 Methods

### 2.1 Study Design

This multicenter retrospective cohort study included fetuses diagnosed with open spinal dysraphism (OSD) across three prenatal care centers. All centers followed a standardized protocol for prenatal diagnosis and postnatal follow‐up. We analyzed cases of postnatally confirmed OSD without hindbrain herniation between 19 and 27 weeks of gestation. The study aimed to characterize prenatal findings, the natural history, and postnatal outcomes by tracking the progression of ventriculomegaly, hindbrain herniation, and motor function from mid‐gestation to the postnatal period. Participants were recruited from University Hospital Gießen and Marburg (UKGM) in Germany, Erasmus MC University Medical Center in Rotterdam, the Netherlands, and Instituto Materno‐Perinatal in Lima, Peru. Initial ethical approval for this study was obtained from the University Hospital Institutional Review Board in Giessen (IRB approval AZ 161/20) and Marburg (IRB approval AZ 23‐280 BO), with subsequent local approval obtained at Erasmus MC (MEC 2023‐ 0462) and Instituto Nacional Materno‐Perinatal Lima (IRB 18–164351).

### 2.2 Patient Selection and Eligibility

We conducted a retrospective review of medical records from pregnancies diagnosed with OSD, including myelomeningocele (MMC) and myeloschisis (MS), between January 2016 and August 2024. The aim was to identify cases presenting without HBH, defined as downward displacement of the cerebellar vermis below the foramen magnum during second‐trimester assessment, as confirmed by ultrasound (US) and magnetic resonance imaging (MRI). Eligibility for study inclusion further required postnatal confirmation of open spinal dysraphism by experienced neurosurgeons or, in cases of pregnancy termination, by fetal pathologists.

All three centers adhered to the MOMS selection criteria for fetal surgery and thus fetal surgery was not offered in the absence of HBH.

### 2.3 Protocols and Evaluation Timeline

Standardized evaluation protocols were implemented across all sites based on institutional guidelines. Routine invasive genetic testing, based on local standards (including karyotype, single nucleotide polymorphism (SNP) Array and exome sequencing (ES)) was offered to all patients presenting for evaluation, along with additional measurements of alpha‐fetoprotein (AFP) and/or acetylcholinesterase (AChE) in more recent cases. In cases where ES was performed, variant interpretation was performed according to the American College of Medical Genetics and Genomics criteria [20]. Participants underwent evaluations at three primary intervals: an initial assessment between 20‐ and 27‐weeks of gestation, a follow‐up around 30 weeks of gestation, and a postnatal evaluation during the neonatal period. When feasible, retrospective chart reviews were performed to evaluate the incidence of shunting beyond the neonatal period.

### 2.4 Prenatal Evaluation

Transabdominal and transvaginal US were conducted to evaluate cranial and cerebral findings and the lesion of these fetuses. This included assessing the typical concavity of the frontal bones (“lemon sign”), the characteristic crescentic shape of the cerebellum in a small posterior fossa (“banana sign”), and measurement of head circumference (HC) and biparietal diameter (BPD) using established nomograms. Anomalies of the corpus callosum were evaluated in all 12 fetuses by MRI or transvaginal ultrasound, and ventricular width was measured following published guidelines [21]. Ventriculomegaly was defined as a lateral ventricle atrial diameter ≥ 10 mm on prenatal ultrasound, with moderate ventriculomegaly classified as less than 15 mm and severe ventriculomegaly classified as 15 mm or more [21, 22]. The assessment of the posterior fossa focused on identifying HBH below the foramen magnum and measuring the CSA, as previously published [23, 24]. The examination of the lesion itself was performed in three orthogonal planes. The anatomic level was defined as the first affected vertebra. Prenatal MRI with a 1.5 T scanner was offered to all participants, although one mother declined, in which case only a transabdominal and transvaginal ultrasound was conducted. Motor function was assessed using the methodology described by Carreras et al., with ultrasound assessment of motor function according to the lower extremity movements: Level Lumbar (L) 1 corresponds to hip flexion, L2 corresponds to hip adduction, L3 corresponds to the knee extension, L4 to knee flexion, L5 to dorsal flexion of the ankle and Sacral (S)1 to plantar flexion of the ankle [25, 26].

### 2.5 Postnatal Evaluation

Postnatal evaluation of HBH was performed using US and MRI. Spinal lesion type was confirmed by an experienced neurosurgeon both after birth and during postnatal surgery. Criteria for shunt placement were according to local standards, with all three centers adhering to the criteria published in the MOMS protocol [1]. In our cohort, functional segmental motor function was assessed after birth through a detailed neurological examination performed by a pediatric neurosurgeon and/or pediatric neurologist within the first 48 h. After discharge, all infants were followed according to each center's standardized protocol. Neurological assessments, including sensory, motor, and cutaneous evaluations of the lower extremities, were conducted at each visit by developmental pediatricians. Motor levels were determined based on the lowest myotomes involved in active motor function. As the follow‐up period was less than 30 months, we did not include ambulatory status as an outcome in this cohort.

### 2.6 Data Collection and Statistical Analysis

For dichotomous, ordinal and categorical variables, data were summarized using frequencies and percentages. In instances where data points were missing, the percentage was calculated based on the subset of the population for which data was available rather than the entire population. The distribution of continuous group data was assessed using a Quantile–Quantile (Q–Q) plot and a Shapiro‐Wilk test. Continuous variables were summarized using either means ± standard deviations or medians with (interquartile ranges) depending on their distribution. The results are presented as descriptive statistics and given the small sample size no comparative statistical testing was performed. All analyses were performed using SPSS version 29.0 software (IBM Corp.).

## 3 Results

### 3.1 Study Population

**FIGURE 1:**
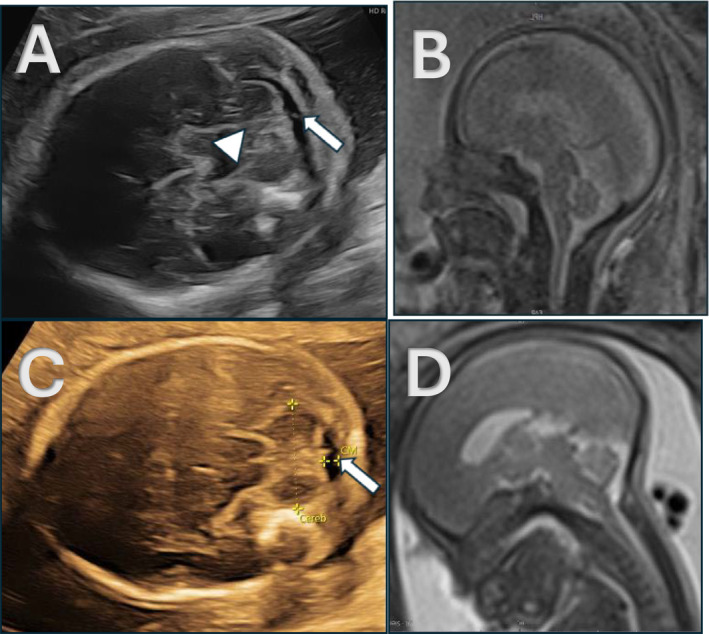
(A–D) Prenatal cranial US and MRI of case numbers 1 and 5. (A, B) Prenatal US and T2‐weighted MRI of fetal brain of case number 1 at 22.9 weeks GA, showing a normal‐shaped cerebellum and a patent fourth ventricle (arrowhead) and cisterna magna (arrow). No HBH is visible on fetal MRI. (C, D) Prenatal US and T2‐weighted MRI of fetal brain of case number 5 at 20.6 weeks GA, showing a normal‐shaped cerebellum and a patent cisterna magna (arrow). No HBH is visible on fetal MRI.

A total of 280 fetuses with MMC and MS were assessed across three fetal medicine centers. This included 196 (70.0%) cases with MMC and 84 (30.0%) cases with MS. Of these, 12 fetuses (4%) had open spinal dysraphism without HBH below the foraman magnum in the second trimester and were included in this study. All included cases were diagnosed with an MMC. Notably, one couple underwent prenatal surgery at another center despite the absence of HBH (Case 1), while another couple opted for termination of pregnancy (Case 5). In both cases, OSD was confirmed intraoperatively or at postmortem examination. Only prenatal findings are reported for these cases, as postnatal follow‐up data were either unavailable or potentially influenced by the effects of fetal surgery (Table 1, Figure: 1 A–D).

**TABLE 1:** Detailed case description on first prenatal evaluation.

	Case 1	Case 2	Case 3	Case 4	Case 5	Case 6	Case 7	Case 8	Case 9	Case 10	Case 11	Case 12
Findings at first evaluation (19–27 weeks)												
Lemon sign	Yes	Yes	Yes	Yes	Yes	No	Yes	No	Yes	No	Yes	Yes
Banana sign	No	No	No	Yes	No	No	No	No	Yes	No	Yes	Yes
BPD ≤ 5th percentile	No	Yes	Yes	Yes	Yes	Yes	Yes	No	No	No	Yes	Yes
TCD ≤ 5th percentile	No	Yes	Yes	Yes	No	Yes	Yes	No	Yes	No	Yes	Yes
Abnormal corpus callosum	No	No	No	No	No	No	No	No	No	No	No	No
Ventricular size (mm)	12.0	7.0	7.6	8.5	11.6	12.6	6.0	7.0	11.0	9.7	9.8	8.2
VM	Yes	No	No	No	Yes	Yes	No	No	Yes	No	No	No
CSA (US^a^/MRI)	72.4°	58.8°	73.3°	58.8°	61.9°	64.0°	68.5°	74.0°	88.7°	/	90.2°*	71.3°*
Anatomic level	L5	L5	L5	L3	L4	L4	L2	S1	L4	L5	L5	L5
Motor level	S1	L4	S1	L1	S1	L5	L3	S1	S1	S1	/	L5
Abnormal foot position	No	Yes	No	Yes	No	No	No	No	No	No	No	No
AFP (MoM)	4.7	4	/	/	5.4	2.4	/	/	/	13.4	4.0	/
AChE	/	/	/	/	Pos.	Pos.	/	/	/	/	/	/

Abbreviations: AChE: acetylcholinesterase, AFP: alpha‐fetoprotein; BPD: biparietal diameter, CSA: clivus‐supraocciput angle; MRI: magnetic resonance imaging; Pos: positive; TCD: transcerebellar diameter; US: ultrasound; VM: ventriculomegaly.

^a^ CSA measured on US.

## 4 Maternal Characteristics

The mean maternal age was 30.4 ± 4.6 years, and the mean body mass index (BMI) was 26.7 ± 5.9. Six cases gave informed consent for invasive genetic testing, identifying a variant of uncertain significance in the TBXT gene (OMIM‐G 601397) (8.3%). Amniotic fluid AFP was measured in all six cases, with all but one exceeding the 2.5 MoM threshold (median 4.4 (3.8) MoM). In one case, AFP was 2.4 MoM, but AChE was positive. AChE was assessed in two out of six cases (17%) and was elevated in both.

### 4.1 Fetal Characteristics

The median gestational age at the baseline ultrasound assessment was 21.0 weeks (3.5). During the initial evaluation, most fetuses presented with a BPD below the 5th percentile (67%) and a transverse cerebellar diameter (TCD) also below the 5th percentile (67%). A lemon sign was observed in 75% of cases, and a banana sign in 33%. The average lateral ventricle diameter of 9.2 ± 2.2 mm. Moderate ventriculomegaly was present in 33% of cases, while no severe cases were noted. The mean CSA was 71.1 ± 10.6°, and no corpus callosum abnormalities were identified. The upper defect level ranged from L2 to S1, with most cases starting at L5 (50%). The prenatal motor level was available in 11 of the 12 fetuses; the motor level was most frequently S1 (55%), with an overall range from L1 to S1. An abnormal foot position was observed in 17% of cases (Table 1).

Third‐trimester ultrasound data were available for nine fetuses, with a mean gestational age of 30.3 ± 1.0 weeks. Only postnatal outcome data were available in cases seven, as no intrauterine follow‐up was conducted. Cases one and five were excluded for the reasons described above. At 30 weeks of GA, HBH was evaluated in 8 out of 10 fetuses (80%) and was observed in two (25%, cases 3 and 6). In the two cases without information on HBH at 30 weeks, HBH was absent after birth—additionally, three fetuses developed ventriculomegaly, which had not been observed during the initial evaluation. The mean ventricular width was 10.1 ± 2.0 mm, as detailed in Table 2.

**TABLE 2:** Follow‐up outcomes of third‐trimester ultrasound and postnatal evaluation.

	Case 2	Case 3	Case 4	Case 6	Case 7	Case 8	Case 9	Case 10	Case 11	Case 12
Third‐trimester outcomes										
Ventricular size (mm)	9.8	7.5	11.8	14.1	/	8.1	9.4	8.7	11.0	10.7
VM	No	No	Yes	Yes	/	No	No	No	Yes	Yes
HBH	No	Yes	/	Yes	/	No	No	No	No	No
Postnatal outcomes										
Motor level after birth	L4	L5	L1	L4	L4	S2	L4	S1	L5	L5
HBH After birth	No	Yes	No	Yes	No	No	Yes	Yes	Yes	No
Shunt in the follow‐up period	No	No	No	Yes	No	No	No	No	Yes	No
Follow‐up duration (months)	24	13	13	1	10	18	22	13	24	6

Abbreviations: HBH: hindbrain herniation, VM: ventriculomegaly.

### 4.2 Postnatal Outcomes

**FIGURE 2:**
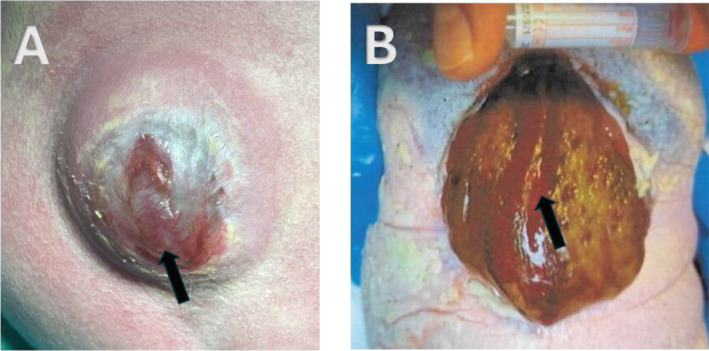
(A, B) Postnatal image of case numbers 3 and 4 showing a non‐neurulated placode (arrow). In Case 3, a broad skin wall with a gradual transition from skin to meninges is visible (Figure 2B copyright Prof. Raymund Horch, Department of Plastic and Hand Surgery, University Hospital Erlangen, Friedrich Alexander University Erlangen‐Nuernberg).

Postnatal follow‐up was available for 10 cases (83%). All lesions displayed a non‐neurulated placode which is a characteristic of an open defect (Figure 2 A,B). In some cases, instead of an abrupt transition between the skin and the meninges, we observed a broad cutaneous rim encompassing the base of the lesion, which gradually transitioned into the meninges and the placode.

The mean follow‐up duration of the remaining 10 cases was 14.4 ± 8.9 months. Among these 10 cases, five (50%) exhibited HBH below the foramen magnum on postnatal MRI, with two having already shown this finding at the 30‐week US. Unlike the typical findings of Chiari II deformity, these cases did not necessarily demonstrate a small posterior fossa or absence of the cisterna magna or fourth ventricle. The shunt rate during the follow‐up period was 2/10 (20%), and both shunts were placed in the neonatal period (up to 28 days postnatally). Both neonates demonstrated HBH on postnatal imaging, with this finding already present in one case during the third trimester.

Motor function levels varied, with most cases having a motor level at L4 (40%) or L5 (30%), ranging from L1 to S2. Motor function levels after birth were equal or better to the anatomic level in 8 fetuses (80%), while 2 fetuses (20%) demonstrated motor levels one or two levels worse. Compared to the initial prenatal assessments, motor function was equal or better in 6 fetuses (67%) and worsened by one to two levels in 3 fetuses (33%). In one fetus (Case 11), prenatal motor function was not assessed (Table 2).

## 5 Discussion

This case series contributes to the understanding of the natural history of OSD without HBH. In our cohort, 4% of all cases assessed for OSD in the second trimester showed no evidence of HBH at that time. Notably, this absence was exclusively observed in cases of MMC. Among this subgroup, HBH developed in 50% of cases during pregnancy. Ventriculomegaly was moderate and infrequent during the first prenatal evaluation. It increased slightly in the third trimester with an overall mild progression. The shunt rate in this subgroup was low (20%) over a mean follow‐up period of 14.4 months. We observed a high rate of motor function impairment already at the first prenatal evaluation in the second trimester. In around a third of cases, postnatal motor function appeared to have attenuated compared with the initial fetal evaluation.

The prevalence of OSD without HBH varies significantly across studies, ranging from 8% to 23% [9, 16, 17, 19].. The discrepancy with our series may be attributed to differences in study design and case ascertainment. Despite all efforts, the differentiation between MMC and other cystic forms of spinal dysraphism (such as Limited Dorsal Myeloschisis) can be challenging and postnatal or intraoperative confirmation is mandatory to confirm the diagnosis [27, 28]. Unlike prior studies, we included only cases with postnatal or intraoperative confirmation of OSD. Additionally, selection bias is not unlikely as all three hospitals are referral centers for fetal surgery, and thus, cases without obvious HBH may not have been referred.

Absence of HBH was observed exclusively in cases of MMC and not in MS, consistent with prior literature indicating that the extent of hindbrain herniation (HBH) inversely correlates with sac size and is more pronounced in flat lesions, suggesting that both the presence and morphological features of the sac may modulate CSF dynamics, potentially leading to a slower and less severe CSF leak [9, 29, 30].

HBH at second‐trimester evaluation was one criterion for fetal surgery as outlined by the MOMS protocol [1]. Fetal surgery improves posterior fossa size and reverses HBH, thereby decreasing HBH‐associated complications, improving CSF dynamics and reducing the need for postnatal CSF diversion [1, 17, 31, 32, 33]. In our cohort without prenatal surgery, the low incidence of postnatal shunt placement suggests limited added benefit of fetal surgery in minimizing shunt dependency in this subgroup. On the other hand, the two fetuses that required a shunt exhibited progressive ventriculomegaly in the third trimester and hindbrain herniation after birth.

There are limited data on the progression of HBH in fetuses without HBH at the initial evaluation. One study reported hindbrain herniation in 1 out of 8 fetuses after birth, while a recent study of 73 fetuses observed a 12% progression rate during pregnancy, leading to adjustments in prenatal management, including the consideration of prenatal surgery, in certain cases [9, 17]. Although the timing of the second evaluation in that study is not specified, it likely occurred before 26 weeks, earlier than in our cohort, where the follow‐up was at 30 weeks. In those expectantly managed, 17% had HBH after birth; however, outcomes were missing in nearly a third of cases. In accordance with our findings, the shunt rate was significantly lower than in fetuses with HBH [9].

MMC without HBH appears to represent a milder phenotype within the spectrum of open spinal dysraphism. Ventriculomegaly, typically occurring in fetuses with open spinal defects, often progresses with gestation; however, in our cohort, it was present in only 33% initially, increasing modestly to 44% in the third trimester, with a mean ventricular width progressing from 9.2 mm at baseline to 10.1 mm around 30 weeks [34, 35, 36, 37]. The CSA averaged 71° in our cohort, an intermediate value between cases with open spinal dysraphism and healthy controls [24]. Furthermore, while corpus callosum anomalies are observed in up to 72% of open spinal dysraphism cases on MRI and expert fetal neurosonography and correlate with more severe ventriculomegaly, no such abnormalities were present in our cohort, suggesting a potentially more favorable neurocognitive prognosis [38, 39, 40, 41, 42]. The explanation for the low incidence is not entirely clear though lesion characteristics such as decreased CSF leakage may play a role [9].

Motor function changes were evident in 45% of cases at 19–27 weeks, a higher percentage compared to other studies, where unimpaired motor function was observed in over 80% [43, 44]. After birth, motor function was equal to or better than the anatomical level in 80% of cases. When comparing the initial prenatal functional evaluation, motor function worsened by one to two levels in 33%. Intrauterine factors, such as prolonged neural placode exposure and mechanical stretching in cystic lesions, may contribute to changes in motor function [45, 46, 47, 48, 49]. However, interpretation of these findings is limited by challenges in prenatal functional assessment, including short observation times and suboptimal fetal positioning. For cases with HBH, there is some evidence that motor function remains stable after prenatal surgery from the time of referral to 12 months of life [43]. Considering these factors, it may be possible that prenatal surgery may also offer benefits for MMC without HBH, though this must be balanced against significant maternal risks.

### 5.1 Strengths and Limitations

A key strength lies in the careful case selection process, which required postnatal confirmation of OSD by neurosurgeons or fetal pathologists and not only prenatal imaging criteria. This methodological rigor minimizes diagnostic ambiguity.

However, the study is limited by a small sample size and a short follow‐up period of less than 30 months, preventing definitive conclusions regarding motor function and ambulatory ability. Additionally, sac size, which may influence motor function and the severity of hindbrain herniation, was not measured [9, 45, 46, 47].

Prospective multicenter studies with larger cohorts, standardized protocols, and extended follow‐up are needed to validate these findings.

## 6 Conclusion

Fetuses with OSD and the absence of HBH in the second trimester demonstrate overall moderate or no ventriculomegaly and show a slow progression in ventricular size. Although 50% exhibited HBH postnatally, there was a low shunt rate. Postnatal motor function was affected, corresponding to the level of vertebral defect and often did not worsen compared with second trimester evaluation. Our findings may aid in decision‐making and prenatal counseling; however, we cannot determine whether prenatal surgery provides a benefit for these lesions, as they have a relatively better outcome compared to MMC with HBH. Further prospective studies are needed to clarify this question.

## Ethics Statement

Initial ethical approval for this study was obtained from the University Hospital Institutional Review Board in Giessen (IRB approval AZ 161/20) and Marburg (IRB approval AZ 23‐280 BO), with subsequent local approval obtained at the other two sites.

## Consent

Because of the retrospective nature of the study, informed consent from the patients was waived as the study analyzed anonymized clinical patient data.

## Conflicts of Interest

The authors declare no conflicts of interest.

## Data Availability

The data used to support this study's findings are available from the corresponding author upon reasonable request.
